# Maternal Prenatal Stress Is Associated With Altered Uncinate Fasciculus Microstructure in Premature Neonates

**DOI:** 10.1016/j.biopsych.2019.08.010

**Published:** 2020-03-15

**Authors:** Alexandra Lautarescu, Diliana Pecheva, Chiara Nosarti, Julie Nihouarn, Hui Zhang, Suresh Victor, Michael Craig, A. David Edwards, Serena J. Counsell

**Affiliations:** a Department of Perinatal Imaging and Health, Centre for Developing Brain, School of Biomedical Engineering and Imaging Sciences, King’s College London, London, United Kingdom; b Department of Forensic and Neurodevelopmental Sciences, Institute of Psychiatry, Psychology and Neuroscience, King’s College London, London, United Kingdom; c Department of Computer Science and Centre for Medical Image Computing, University College London, London, United Kingdom; d National Female Hormone Clinic, South London and Maudsley National Health Service Foundation Trust, London, United Kingdom

**Keywords:** Diffusion tensor imaging, Neonatal brain, Prematurity, Stressful life events, Uncinate fasciculus, White matter

## Abstract

**Background:**

Maternal prenatal stress exposure (PNSE) increases risk for adverse psychiatric and behavioral outcomes in offspring. The biological basis for this elevated risk is poorly understood but may involve alterations to the neurodevelopmental trajectory of white matter tracts within the limbic system, particularly the uncinate fasciculus. Additionally, preterm birth is associated with both impaired white matter development and adverse developmental outcomes. In this study we hypothesized that higher maternal PNSE was associated with altered uncinate fasciculus microstructure in offspring.

**Methods:**

In this study, 251 preterm infants (132 male, 119 female) (median gestational age = 30.29 weeks [range, 23.57–32.86 weeks]) underwent brain magnetic resonance imaging including diffusion-weighted imaging around term-equivalent age (median = 42.43 weeks [range, 37.86–45.71 weeks]). Measures of white matter microstructure were calculated for the uncinate fasciculus and the inferior longitudinal fasciculus, a control tract that we hypothesized was not associated with maternal PNSE. Multiple regressions were used to investigate the relationship among maternal trait anxiety scores, stressful life events, and white matter microstructure indices in the neonatal brain.

**Results:**

Adjusting for gestational age at birth, postmenstrual age at scan, maternal age, socioeconomic status, sex, and number of days on parenteral nutrition, higher stressful life events scores were associated with higher axial diffusivity (β = .177, *q* = .007), radial diffusivity (β = .133, *q* = .026), and mean diffusivity (β = .149, *q* = .012) in the left uncinate fasciculus, and higher axial diffusivity (β = .142, *q* = .026) in the right uncinate fasciculus.

**Conclusions:**

These findings suggest that PNSE is associated with altered development of specific frontolimbic pathways in preterm neonates as early as term-equivalent age.

SEE COMMENTARY ON PAGE 487; SEE ALSO VIDEO CONTENT ONLINE

Maternal prenatal stress exposure (PNSE) represents a global public health problem 1, 2, 3, 4 and affects 10% to 35% of children worldwide (5). In particular, exposure to stressful life events and prenatal maternal anxiety has been associated with an increased risk for a range of adverse behavioral outcomes in offspring. These include more crying and/or fussing (6), anxiety disorders (7), externalizing behavior (8), attention-deficit/hyperactivity disorder (9), and conduct disorders (10). Furthermore, these changes can lead to a transgenerational cycle of adaptations of brain function and behavior (11). However, the biological mechanism(s) that translate maternal PNSE into behavioral changes in offspring remain poorly understood. One potential mechanism involves disruption of the neurodevelopment of specific white matter tracts within the limbic system (12).

White matter development can be assessed in vivo using diffusion tensor imaging (DTI) (13), which characterizes water molecular motion in tissue and provides objective metrics including fractional anisotropy ([FA], a measure of the directional dependence of water diffusion); mean diffusivity ([MD], the magnitude of water diffusion within brain tissue); radial diffusivity ([RD], an estimate of the magnitude of diffusion perpendicular to the direction of fibers); and axial diffusivity ([AD], the estimated magnitude of diffusion parallel to the direction of fibers). DTI tractography is a noninvasive neuroimaging technique that can be used to delineate the trajectories of white matter fibers and enables tract-specific measures to be obtained, allowing comparison of corresponding fasciculi between individuals.

PNSE has been linked to abnormal neurodevelopment of a number of brain regions including the limbic system and prefrontal cortex, in both animal 14, 15, 16, 17 and human 18, 19 studies. Previous DTI studies in neonates exposed to PNSE have, for example, reported reduced FA and increased MD, RD, and AD in multiple fiber bundles within the limbic system 20, 21, 22. The most consistently reported finding involves altered development of white matter fibers connecting the amygdala with the prefrontal cortex, which are contained within the uncinate fasciculus 19, 23, 24. This is a white matter association tract that has been implicated in several neurodevelopmental and psychiatric disorders (25), specifically anxiety disorders and early-life stress 26, 27, 28, 29, 30.

Preterm birth affects approximately 11% of global live births and is associated with adverse neuropsychiatric and developmental outcomes 31, 32, 33, 34, 35, 36. A number of studies have focused on investigating the relationship between brain development and these adverse outcomes 37, 38, 39, with aberrant white matter microstructural development 38, 40, 41, 42 being commonly reported. However, it is important to also assess the role that early adverse experiences may have in moderating these associations. Some studies have suggested an increased risk of preterm birth in women experiencing a high number of stressful life events or increased anxiety 43, 44, 45, 46. To our knowledge, however, no studies have examined the relationship between PNSE and white matter microstructure in infants born prematurely.

In this study, we assessed the relationship between maternal PNSE and white matter microstructure of the uncinate fasciculus in a large sample of premature neonates. We hypothesized that higher scores on maternal stressful life events and trait anxiety would be associated with decreased FA and increased RD, AD, and MD in the uncinate fasciculus.

## Methods and Materials

### Participants

A total of 511 premature infants (born before 33 weeks of gestational age) took part in the Evaluation of Preterm Imaging Study (ePRIME), a randomized control trial that investigated the effect of having a brain magnetic resonance (MR) imaging or ultrasound scan at term-equivalent age on parental anxiety (47). As part of this study, data were collected on maternal anxiety (State-Trait Anxiety Inventory [STAI]), stressful life events, demographic data, and perinatal clinical risk factors. MR images were reviewed by a perinatal neuroradiologist. Women who reported alcohol and drug abuse during pregnancy (*n* = 6) and cases with major focal lesions such as periventricular leukomalacia, hemorrhagic parenchymal infarction, and other ischemic or hemorrhagic lesions (*n* = 40) were excluded from analysis (Supplemental Table S1). In the case of multiparous pregnancies, only 1 infant from a twin and/or triplet pregnancy was included in this study (selected at random). From the remaining sample, DTI data, demographics, and both STAI and stressful life events data were available for 251 mother-infant dyads. Descriptive statistics are presented in Table 1 (for infant characteristics) and Table 2 (for maternal characteristics).

**Table 1:** Infant Obstetric and Sociodemographic Characteristics

Infant Characteristics	Values
Gestational Age at Birth, Weeks, Median (Range)	30.29 (23.57–32.86)
Postmenstrual Age at Scan, Weeks, Mean ± SD	42.21 ± 1.64
Total Parenteral Nutrition, Days, Median (Range)	6.00 (0–59)
Total Ventilation, Days, Median (Range)	0 (0–33)
Total Number of Pregnancy Complications, Median (Range)	1 (0–5)
Birth Weight, g, Median (Range)	1290.00 (572.00–2600.00)
Head Circumference at Birth, cm, Mean ± SD	28.94 ± 3.05
Sex, *n* (%)	
Male	132 (52.6)
Female	119 (47.4)

Mean and SD are reported for normally distributed data; median and range are reported for nonnormally distributed data.

**Table 2:** Maternal Sociodemographic Characteristics

Maternal Characteristics	Values
Maternal Age, Years, Mean ± SD	33.35 ± 5.83
Socioeconomic Status, Median (Range)	17.06 (1.73–60.58)
Maternal Trait Anxiety, Median (Range)	36.00 (20.00–68.00)
Stressful Life Events Score, Median (Range)	53.00 (0–270.00)
Maternal Ethnicity, *n* (%)	
White British or Irish	97 (38.7)
Other white background	38 (15.1)
Black or Black British	49 (19.6)
Mixed race	4 (1.6)
Asian or Asian British	54 (21.5)
Other ethnicity group	6 (2.4)
Not reported	3 (1.2)
Maternal Age on Leaving Formal Education, *n* (%)	
16 years or less	22 (8.8)
17–19 years	35 (13.9)
19+ years	180 (71.7)
Still in full-time education	9 (3.6)
Not reported	5 (2.0)

Mean and SD are reported for normally distributed data; median and range are reported for nonnormally distributed data.

Ethical approval was obtained from the Hammersmith and Queen Charlotte’s Research Ethics Committee (09/H0707/98).

### Trait Anxiety

The STAI (48) was administered at the time of the scan. There are 2 subscales within this measure; State Anxiety measures the current level of anxiety, with questions referring to how participants feel “right now,” while Trait Anxiety (STAI-TR) measures the relatively stable tendency to be prone to anxiety, with questions referring to how participants feel “in general” (48). We restricted our analysis of anxiety to STAI-TR, as it extends to the period before birth. For STAI-TR, missing values were imputed for participants (*n* = 32) who had missing values on a maximum of 10% of questions (*n* = 28 missing 1 answer out of 20, *n* = 4 missing 2 answers out of 20; see the Supplement). Missing data were imputed by calculating the average score for the questions that were answered and imputing this value.

### Stressful Life Events

All mothers completed a questionnaire measuring the number of stressful life events they experienced in the year prior to the study visit (e.g., “Arguments with your partner increased”). The questionnaire was adapted from the Avon Longitudinal Study of Parents and Children (49) and was administered to include only yes-no answers. To obtain a continuous score, stressful life events were ranked according to severity based on the Social Readjustment Rating Scale (50). The final score was then calculated for each mother to represent a sum of the severity scores for the stressful life events she experienced (Supplemental Table S2). Face validity for this adapted questionnaire was established through examination by a consultant psychiatrist (MCC) with experience in anxiety and mood disorders.

There were no missing data on the stressful life events questionnaire.

### MR Imaging

Three-dimensional magnetization prepared rapid acquisition gradient echo (repetition time: 17 ms; echo time: 4.6 ms; flip angle: 13°; slice thickness: 0.8 mm; in-plane resolution: 0.82 × 0.82 mm), T2-weighted turbo spin echo (repetition time: 8670 ms; echo time: 160 ms; flip angle: 90°; slice thickness: 2 mm; in-plane resolution: 0.86 × 0.86 mm), and single shot echo planar DTI (repetition time: 7536 ms; echo time: 49 ms; flip angle: 90°; slice thickness: 2 mm; in-plane resolution: 2 x 2 mm, 32 noncollinear gradient directions, *b* value of 750 s/mm^2^, 1 non-diffusion-weighted image, *b* = 0) were acquired on a Philips 3T (Philips Medical Systems, Best, The Netherlands) MR system sited on the neonatal intensive care unit using an 8-channel phased array head coil.

All examinations were supervised by a pediatrician experienced in MR imaging. Parents were offered the option of having their infant sedated with oral chloral hydrate (25–50 mg/kg) prior to scanning (219 infants were sedated). Pulse oximetry, temperature, and electrocardiography were monitored throughout the scan and ear protection was used, comprising earplugs molded from a silicone-based putty (President Putty; Coltene Whaledent, Mahwah, NJ) placed in the external auditory meatus and neonatal earmuffs (MiniMuffs; Natus Medical Inc., San Carlos, CA).

### DTI Analysis

Diffusion-weighted images were visually inspected in 3 orthogonal planes for the presence of motion artifact, and corrupt diffusion-weighted volumes were excluded before tensor fitting. Seventy-seven datasets had at least 1 volume removed (median: 0 [range, 0–8]). Image processing and data analysis were performed using FMRIB's Diffusion Toolbox (version 3.0) and DTI-TK (version 2.3.1; http://.dti-tk.sourceforge.net) (51). For each infant the diffusion-weighted images were registered to their native b0 image and corrected for differences in spatial distortion using eddy correct. Nonbrain tissue was removed with FSL’s BET [version 2.1; http://fsl.fmrib.ox.ac.uk/fsl/fslwiki/BET 52, 53].

Diffusion tensors were calculated on a per-voxel basis, using a simple least-squares fit of the tensor model to the diffusion data. From this the tensor eigenvalues describing the diffusion strength in the primary, secondary, and tertiary diffusion directions were obtained. AD, RD, MD, and FA maps were calculated for each subject.

**Figure 1:**
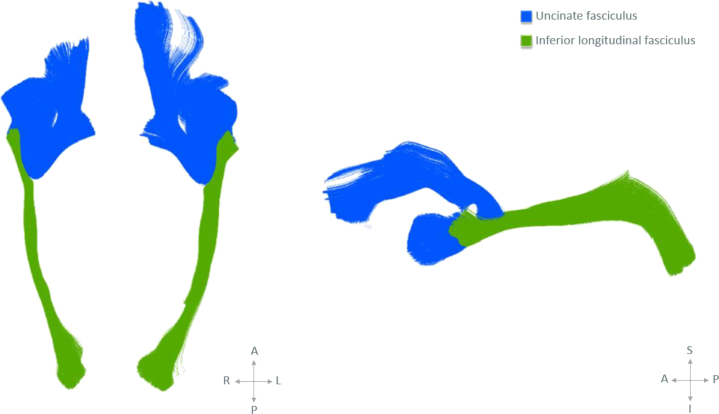
Diffusion tensor imaging tractography of the uncinate fasciculus (blue) and inferior longitudinal fasciculus tract (green) in axial and sagittal planes (left to right). A, anterior; I, inferior; L, left; P, posterior; R, right; S, sagittal.

**Figure 2:**
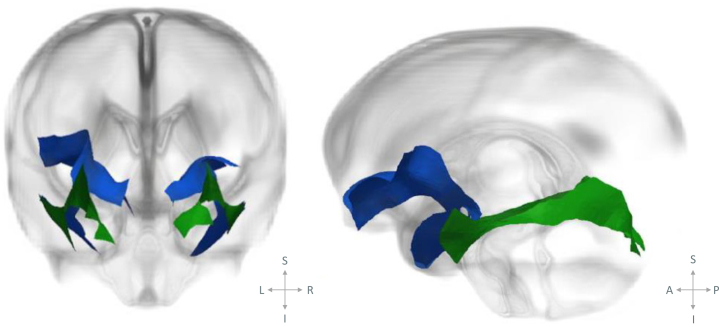
“Glass brain” illustrations showing the skeletonized versions of the uncinate fasciculus (blue) and inferior longitudinal fasciculus (green) medial surface overlaid on the template radial diffusivity image, presented in coronal and sagittal planes (left to right). See the Supplemental Video for 3-dimensional data visualization. A, anterior; I, inferior; L, left; P, posterior; R, right; S, sagittal.

DTI measures were derived for each subject using tract-specific analysis (54) as described in Pecheva *et al.* (55). Briefly, a study-specific template was created by registering all subjects together to create an iteratively refined average tensor image (54). Following registration, tracts of interest were delineated within the template using deterministic tractography based on the FACT approach (56) (part of DTI-TK) and manually drawn regions of interest (57). We delineated the left and right uncinate fasciculus as well as a “nonlimbic” control tract, the inferior longitudinal fasciculus. The inferior longitudinal fasciculus connects the occipital cortex to the temporal lobe (58), and it was selected as a control tract as it shares a termination point with the uncinate fasciculus but has not been implicated in social and emotional behavior (58). This tract has been used as a control tract in previous studies focusing on children who were exposed to maternal stress (12). From the tractography results, the tract-specific analysis medial representation model was used to create tractwise white matter skeletons of the uncinate fasciculus and inferior longitudinal fasciculus (Figure 1). Each white matter skeleton comprises a medial surface (Figure 2) and tract boundary (59). Diffusion data from each subject were projected onto the skeleton by searching for the tensor with the highest FA value along the unit normal from each point on the skeleton to the tract boundary, as described in Pecheva *et al.* (55). Whole tract average AD, RD, MD, and FA values were calculated for each subject (Supplemental Table S3).

### Statistical Analysis

Statistical analyses were performed using SPSS version 24 (IBM Corp., Armonk, NY), graphs were created with R package ggplot2 (R Foundation for Statistical Computing, Vienna, Austria) 60, 61, and figures were created with ParaView (62). Multiple linear regressions were used to examine associations between maternal anxiety (STAI-TR) and stressful life events with diffusion properties in the left and right uncinate fasciculus (FA, apparent diffusion coefficient, AD, RD) in preterm neonates. Assumptions for multiple regression were met (i.e., residuals were normally distributed, no multicollinearity, homoscedastic data), and there were no missing data in any of the variables included in the model. For each regression, one diffusion measure was considered as an outcome variable, with STAI-TR and stressful life events used as predictors in the same model. Correction for multiple comparisons was done using the Benjamini and Hochberg false discovery rate correction.

The relationship between potential covariates and variables of interest was assessed through bivariate Pearson’s correlations (Table 3). We assessed the following relevant perinatal clinical covariates: gestational age at birth (GA), postmenstrual age at scan (PMA), birth weight, occipitofrontal circumference at birth, socioeconomic status (SES) assessed with the Carstairs Index (63), maternal age, maternal education, total number of pregnancy complications, number of days on total parenteral nutrition (TPN), and number of days on mechanical ventilation. The covariates that remained in the model were GA, PMA, TPN (based on associations with uncinate fasciculus microstructure) (Table 3), SES, sex, and maternal age (based on previous literature). Birth weight was not included as a covariate, as it was very highly correlated with GA (*r* = .76, *p* < .001) and would have introduced multicollinearity in the regression analysis. The number of days on ventilation was not included as a covariate, as it was highly correlated with TPN (*r* = .61, *p* < .001), and both measures provide information on the health status of infants. There was no significant difference between male and female infants on any of the variables included in the model.

**Table 3:** Relationships Between Potential Covariates and Microstructural Properties of the Left and Right Uncinate Fasciculus

	L-UF				R-UF			
	FA	MD	AD	RD	FA	MD	AD	RD
GA	*r* = −.046 *p* = .463	*r* = .213 *p* = .001^a^	*r* = .239 *p* < .001^a^	*r* = .196 *p* = .002^a^	*r* = −.103 *p* = .105	*r* = .199 *p* = .002^a^	*r* = .208 *p* = .001^a^	*r* = .191 *p* = .002^a^
PMA	*r* = .586 *p* < .001^a^	*r* = −.642 *p* < .001^a^	*r* = −.570 *p* < .001^a^	*r* = −.661 *p* < .001^a^	*r* = .625 *p* < .001^a^	r = −.658 *p* < .001^a^	*r* = −.595 *p* < .001^a^	*r* = −.674 *p* < .001^a^
Birth Weight	*r* = −.001 *p* = .986	*r* = .192 *p* = .002^a^	*r* = .231 *p* < .001^a^	*r* = .1720 *p* = .007^a^	*r* = −.055 *p* = .388	*r* = .175 *p* = .005^a^	*r* = .193 *p* = .002^a^	*r* = .163 *p* = .010^a^
Head Circumference at Birth	*r* = .051 *p* = .457	*r* = .009 *p* = .892	*r* = .024 *p* = .723	*r* = .002 *p* = .972	*r* = .022 *p* = .747	*r* = −.019 *p* = .777	*r* = −.021 *p* = .759	*r* = −.018 *p* = .790
Socioeconomic Status	*r* = .037 *p* = .559	*r* = −.055 *p* = .388	*r* = −.050 *p* = .430	*r* = −.056 *p* = .380	*r* = .082 *p* = .194	*r* = −.057 *p* = .366	*r* = −.034 *p* = .588	*r* = −.066 *p* = .295
Maternal Age	*r* = .101 *p* = .112	*r* = −.028 *p* = .654	*r* = −.006 *p* = .992	*r* = −.038 *p* = .550	*r* = .073 *p* = .247	*r* = −.043 *p* = .501	*r* = −.035 *p* = .585	*r* = −.045 *p* = .474
Maternal Education	*r* = −.054 *p* = .395	*r* = .092 *p* = .149	*r* = .088 *p* = .168	*r* = .092 *p* = .150	*r* = −.069 *p* = .283	*r* = .063 *p* = .323	*r* = .055 *p* = .387	*r* = .065 *p* = .307
Pregnancy Complications	*r* = .030 *p* = .633	*r* = .024 *p* = .705	*r* = .041 *p* = .515	*r* = .016 *p* = .806	*r* = .040 *p* = .526	*r* = −.017 *p* = .788	*r* = −.010 *p* = .872	*r* = −.020 *p* = .755
Days TPN	*r* = .052 *p* = .412	*r* = −.150 *p* = .017^a^	*r* = −.163 *p* = .010^a^	*r* = −.142 *p* = .025^a^	*r* = .088 *p* = .163	*r* = −.155 *p* = .014^a^	*r* = −.156 *p* = .013^a^	*r* = −.152 *p* = .016^a^
Days Ventilation	*r* = .036 *p* = .574	*r* = −.183 *p* = .004^a^	*r* = −.206 *p* = .001^a^	*r* = −.169 *p* = .007^a^	*r* = .086 *p* = .175	*r* = −.146 *p* = .020^a^	*r* = −.144 *p* = .022^a^	*r* = −.145 *p* = .022^a^

AD, axial diffusivity; FA, fractional anisotropy; GA, gestational age at birth; L, left; MD, mean diffusivity; PMA, postmenstrual age at scan; R, right; RD, radial diffusivity; TPN, total parenteral nutrition; UF, uncinate fasciculus.

a Results significant at *p* < .05.

## Results

### Demographics

A total of 251 infants (132 male, 119 female) born prematurely were scanned at term-equivalent age. Demographic data are presented in Table 1 (for infant characteristics) and Table 2 (for maternal characteristics). Additional information is presented in Supplemental Table S4.

The number of stressful life events experienced by mothers ranged between 0 and 7 (median = 1 [interquartile range = 1–2]). This included mothers who had experienced no events (*n* = 36), 1 event (*n* = 90), 2 events (*n* = 66), 3 events (*n* = 33), 4 events (*n* = 16), 5 events (*n* = 5), 6 events (*n* = 4), and 7 events (*n* = 1). The stressful life event scores were calculated for each participant based on the severity of experienced events (mean = 68 [range, 0–270]). Using Spearman’s correlation, scores on the stressful life events measure did not correlate with trait anxiety (*r* = .05, *p* = .373).

### Stressful Life Events

#### Associations Between Maternal Stressful Life Events and Uncinate Fasciculus Properties

**Figure 3:**
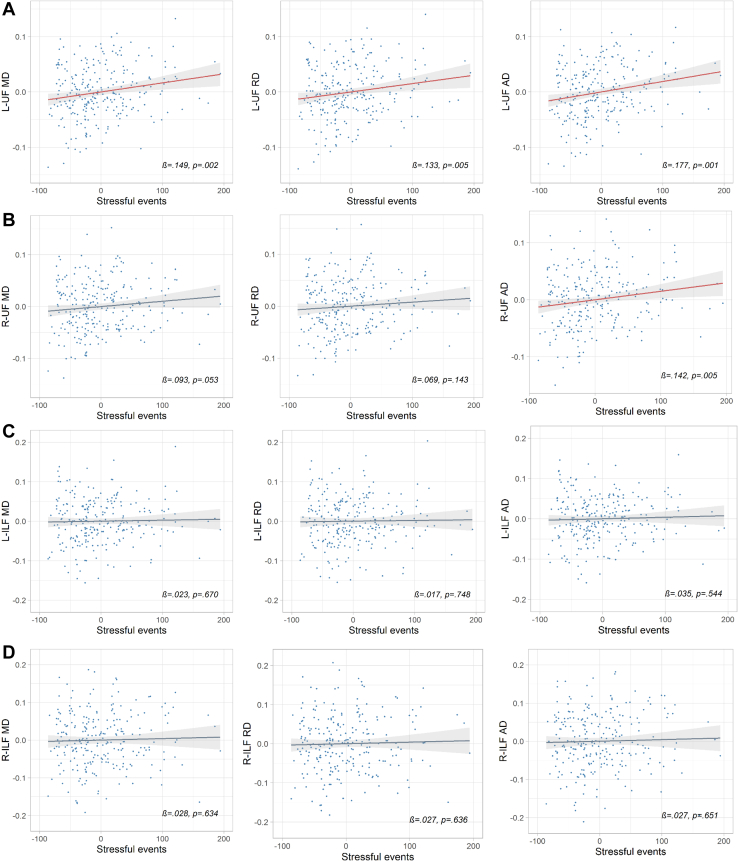
Partial regression scatterplots showing the relationships between stressful life events and mean diffusivity (MD), axial diffusivity (AD), and radial diffusivity (RD) in **(A)** left (L) uncinate fasciculus (UF), **(B)** right (R) uncinate fasciculus, **(C)** left inferior longitudinal fasciculus (ILF), and **(D)** right inferior longitudinal fasciculus, while holding the other predictors constant (i.e., gestational age at birth, postmenstrual age at scan, socioeconomic status, total parenteral nutrition, maternal age, sex). Points on the scatterplot represent residuals and the regression line includes standard error bars. Relationships that were statistically significant are shown in red. β, standardized beta; *p*, significance level before correction for multiple comparisons.

After controlling for GA, PMA, SES, TPN, sex, and maternal age, and after correcting for multiple comparisons, maternal stressful life events were associated with infant left uncinate fasciculus AD (standardized β = .177, *q* = .007, whole-model *R*^2^ = .37), RD (standardized β = .133, *q* = .026, whole-model *R*^2^ = .46), and MD (standardized β = .149, *q* = .012, whole-model *R*^2^ = .44), as well as right uncinate fasciculus AD (standardized β = .142, *q* = .026, whole-model *R*^2^ = .39). Figure 3 shows scatter plots of these relationships, while Table 4 (and Supplemental Table S5) provide more detailed information on the regression models. The only other variable that was associated with uncinate fasciculus microstructure after correction for multiple comparisons was postmenstrual age (*q* < .001). Partial regression scatterplots for nonsignificant relationships are reported in Supplemental Figure S1.

**Table 4:** Associations Between Stress and/or Anxiety and Uncinate Fasciculus Microstructural Properties

Regression	*R*^2^	Adj *R*^2^	*F*	Stressful Events						STAI-TR					
				*B*	β	*t*	*p*	*q*	95% CI	*B*	β	*t*	*p*	*q*	95% CI
L-UF FA	.375	.354	18.113	−0.000005	−.018	−0.344	.731	.820	−.000034 to .000024	0.00017	.110	2.129	.034	.145	.000013 to .00033
R-UF FA	.414	.394	21.346	0.000016	.054	1.088	.278	.467	−.000013 to .000046	0.000073	.045	0.891	.374	.514	−.000089 to .00023
L-UF MD	.448	.429	24.509	0.00016	.149	3.095	.002	.012^a^	.000061 to .00027	−0.00039	−.064	−1.315	.190	.419	−.00097 to .00019
R-UF MD	.456	.438	25.362	0.00011	.093	1.944	.053	.199	−.000001 to .00022	−0.00062	−.098	−2.018	.045	.180	−.0012 to −.000015
L-UF AD	.377	.356	18.280	0.00019	.177	3.464	.001	.007^a^	.000082 to .00030	−0.00023	−.040	−0.781	.436	.545	−.0008 to .00035
R-UF AD	.391	.370	19.385	0.00015	.142	2.804	.005	.026^a^	.000047 to .00027	−0.00067	−.112	−2.18	.030	.137	−.0012 to −.000065
L-UF RD	.467	.449	26.473	0.00015	.133	2.812	.005	.026^a^	.000047 to .00026	−0.00046	−.073	−1.535	.126	.350	−.0010 to .00013
R-UF RD	.472	.455	27.063	0.000086	.069	1.469	.143	.366	−.000029 to .00020	−0.00060	−.089	−1.876	.062	.220	−.0012 to .00003

Results from multiple regression analyses showing the model fit, as well as associations between stress and/or anxiety and uncinate fasciculus microstructural properties. The other covariates in the regression model are gestational age at birth, postmenstrual age at scan, maternal age, sex, socioeconomic status, and days on parenteral nutrition (see the Supplement).

AD, axial diffusivity; Adj, adjusted; *B*, unstandardized beta coefficient; β, standardized beta coefficient; CI, confidence interval; FA, fractional anisotropy; L, left; MD, mean diffusivity; *q*, corrected *p* value using Benjamini-Hochberg false discovery rate correction; R, right; RD, radial diffusivity; STAI-TR, State Trait Anxiety Inventory–Trait Anxiety; UF, uncinate fasciculus.

a Significant results at *p* < .05.

#### Associations Between Maternal Stressful Life Events and Inferior Longitudinal Fasciculus Properties

To determine whether these results are specific to the uncinate fasciculus tract, the above analyses were repeated for the control tract, the inferior longitudinal fasciculus. Neither maternal stressful life events nor trait anxiety predicted diffusion properties in the left or right inferior longitudinal fasciculus (Supplemental Figure S1, Supplemental Table S6).

### Maternal Trait Anxiety

#### Associations Between Maternal Trait Anxiety and White Matter Microstructure

There was no significant relationship between maternal trait anxiety and uncinate fasciculus microstructural properties (Table 4) or inferior longitudinal fasciculus properties (Supplemental Table S6).

### Sensitivity Analyses

There was no association between infant sex and any of the dependent variables.

To check the reliability of the adapted stressful life events scale, we repeated the analyses detailed above excluding the items that did not have a direct equivalent in the Holmes and Rahe scale from the total score (“Your house was burgled,” “Your partner lost his job,” “Your partner was in trouble with the law,” “You took an examination,” and “Your partner had problems at work”). The pattern of results remained the same as when these items were included.

To check the robustness of the results, we repeated our analyses accounting for 1) imputed data for STAI-TR, 2) outliers, 3) postnatal age, 4) ethnicity, 5) multiple births, 6) days on ventilation, 7) emergency cesarean section, 8) intrauterine growth restriction, 9) pregnancy-induced hypertension, 10) larger sample, and 11) age range. The relationship between stressful life events and uncinate fasciculus microstructure retained significance (see the Supplement).

## Discussion

Preterm birth is associated with a range of adverse psychiatric and neurodevelopmental outcomes. To our knowledge, this is the first study examining the relationship between maternal PNSE and brain microstructure in preterm neonates. Our findings suggest that maternal PNSE is associated with alterations in the offspring’s uncinate fasciculus tract as early as term-equivalent age. More specifically, we found that increases in PNSE were associated with higher diffusivity (higher MD, AD, and RD) in the uncinate fasciculus when controlling for GA, PMA, sex, SES, maternal age, and number of days on parenteral nutrition.

The limbic system contains 3 distinct, but partially overlapping, functional networks. These include the dorsomedial default mode, hippocampal-diencephalic-retrosplenial, and temporo-amydala-orbitodrontal networks (64). The uncinate fasciculus is the main tract within the latter network and runs from the anterior part of the temporal lobe, parahippocampal gyrus, uncus, and amygdala to the orbital and polar frontal cortex (64). Abnormal microstructural organization of this tract in children and adults has been associated with a range of outcomes including antisocial behavior 65, 66, autism spectrum disorder 67, 68, anxiety (26), mood disorders 69, 70, obsessive-compulsive disorder (71), and vulnerability to stress (72) and has been observed in children exposed to early adverse experiences such as previous institutionalization 73, 74.

Recent studies provide evidence that the developing white matter is vulnerable to maternal prenatal adversity. Reduced FA in white matter areas including the uncinate fasciculus has been observed in infants of highly anxious mothers 21, 75. Dean *et al.* (20) reported higher diffusivity (increased MD, RD, and AD) in the right frontal white matter of term infants born to mothers experiencing high prenatal symptoms of depression and anxiety.

The reasons for our findings of a relationship between the microstructure of the uncinate fasciculus and PNSE, but not trait anxiety, remain unclear. A number of factors may account for this finding. A recent study into the validity of the STAI in the perinatal period suggests that the mean STAI-TR score in our sample was well below the cutoff range associated with clinically diagnosable DSM-IV anxiety disorder (76). Furthermore, stressful life events and trait anxiety may have different biological correlates (77), such as distinctive inflammatory responses with the transmission of specific cytokines across the placenta, with differential effect on neurodevelopment (78). Furthermore, while maternal anxiety can be a common proxy for stress, experiencing stressful life events during pregnancy does not always coincide with elevated scores on anxiety scales (1). Previous studies reporting associations between maternal antenatal anxiety and infant brain development have focused on state, rather than trait, anxiety (20) or a combined score of state and trait anxiety (21), while those focusing on trait anxiety alone reported no significant associations with brain development (18).

Although the precise mechanisms linking PNSE with neurodevelopmental outcomes in offspring have yet to be determined, research suggests that it may lead to changes in hormones and neurotransmitters in utero (79). This is supported by findings suggesting that maternal cortisol can pass through the placenta (80) and that infants born to mothers who experienced a mood disorder during pregnancy show increased cortisol and norepinephrine, as well as decreased dopamine and serotonin (81). These hormones and neurotransmitters have an essential role in neurogenesis, neuronal differentiation, apoptosis, and synaptogenesis (82), and thus disruption to their normal functioning during critical early-development time periods can lead to changes in brain development, which in turn can lead to adverse neurodevelopmental and behavioral outcomes (83). Animal research has provided support for this, as studies of in utero stress exposure in guinea pigs reported an association between PNSE and reactive astrocyte expression in the hippocampus and subcortical white matter (84), as well as a delay in gamma-aminobutyric acidergic cell number and maturation in the medial frontal cortex and hippocampus, which was further associated with inhibited and anxiety-like behaviors. Furthermore, elevated PNSE has been shown to increase levels of proinflammatory markers across pregnancy (85), which has been linked to decreased FA in the uncinate fasciculus of newborn offspring and decreased cognition at 12 months of age (86). In addition, PNSE is associated with physiological changes including alterations in fetal heart rate (87). Indeed, a recent study assessing structural and functional connectivity in infants exposed to maternal depression suggested that alterations in fetal heart rate may influence the development of the amygdala-prefrontal circuit (88).

PNSE may also affect offspring through epigenetic mechanisms such as DNA methylation and histone modification (89). It is thus likely that the relationship between PNSE and infant white matter microstructure observed in our study is a consequence of the interplay between in utero exposure with genetic and epigenetic mechanisms.

Differences in microstructural properties of white matter tracts are typically explained in relation to differences in myelination. However, myelination in the uncinate fasciculus and inferior longitudinal fasciculus commences in the third postnatal month 90, 91, 92, 93, and thus the differences observed in this study are unlikely to occur as a result of differences in myelination. The elevated diffusivity in the uncinate fasciculus observed here is likely to involve a combination of elevated brain water content, decreases in axon density, increased membrane permeability, and impaired oligodendrocyte proliferation and maturation 94, 95. Reductions in fractional anisotropy are generally related to increases in radial diffusivity or reductions in axial diffusivity (96). The reason we did not observe changes in measured FA in relation to maternal prenatal stress exposure in this study is presumably because we observed an increase in both RD and AD associated with maternal prenatal stress exposure.

Preterm infants in our study were scanned at term-equivalent age and thus were more likely exposed to suboptimal nutrition, ventilation, and other early-life stressors than term-born neonates were. Furthermore, premature birth is known to be associated with altered white matter development 97, 98, 99. However, in this study, we accounted for immaturity at birth and illness severity, and thus these results suggest that prenatal stress may affect the development of white matter in the uncinate fasciculus, above and beyond these additional exposures considered adverse to brain development that are associated with premature birth.

To our knowledge, this represents the largest sample in studies of prenatal stress exposure and infant brain development, as well as the first study to investigate this relationship in a preterm sample. In a recent study by Benavente-Fernández *et al.* (100), the association between brain injury and cognitive outcomes in a sample of children born preterm (24–32 weeks GA) was mediated by maternal SES. Similarly, it is possible that exposure to maternal prenatal stress may exacerbate the risk for negative outcomes in preterm-born children. Future research including term-born control infants is needed to further clarify the nature of this relationship to develop potential interventions that may dampen or reverse the effects of early adversity.

A limitation of our study is that our measure of stressful life events was adapted from a validated questionnaire. However, our results are in line with existing literature on stressful life events and early brain development. Moreover, our measure of life events covers 1 year prior to the scanning session, which includes several months prior to conception. However, Scheinost *et al.* (1) suggested that preconception stress may shape prenatal stress levels and that the cumulative impact of preconception and prenatal stress levels should be considered in research. Although our measures are retrospective, several studies have suggested considerable stability in self-reported anxiety during the perinatal period 76, 101 and accurate recall of pregnancy- and birth-related events 102, 103. A further limitation of this study is the lack of information regarding maternal mental health (especially depression) and use of psychotropic medication, as these have previously been associated with adverse outcomes (1). There is a need for future studies to conduct more comprehensive assessments of maternal psychopathology in the perinatal period. In addition, our study was hypothesis based, focusing on prenatal stress exposure and white matter microstructure in the uncinate fasciculus in offspring. Maternal mental health problems, most notably prenatal depressive symptoms, have been associated with altered microstructure in the cingulum in offspring (104). To look at the wider limbic and association pathways, future prospective studies combining a comprehensive assessment of maternal mental health and with more exploratory whole-brain connectomic approaches [e.g., network-based statistics (105)] have the potential to elucidate specific relationships between a range of prenatal stressors and white matter microstructure across the limbic system and association pathways, while minimizing multiple comparison problems that can arise when comparing a large range of pathways.

Although impairments in uncinate fasciculus microstructure have been associated with behavioral and/or psychiatric outcomes in childhood and/or adolescence in term-born populations (25), it is important to understand whether these findings are observed in preterm-born children. Future studies assessing the relationship between uncinate fasciculus development and subsequent behavioral disorders in this population are required.

In conclusion, we provide what we believe is the first evidence that prenatal stress exposure is associated with altered development of the uncinate fasciculus in premature neonates. These findings add to a growing set of studies implicating maternal prenatal stress in early brain development and suggest that changes in white matter microstructure may be present as early as term-equivalent age.

## Supplementary Material

Supplemental Video

Key Resources Table
