# Supplementary material for: Maternal Prenatal Stress Is Associated With Altered Uncinate Fasciculus Microstructure in Premature Neonates
Source: Biol Psychiatry. 2020 Mar 15;87(6):559–69. doi: 10.1016/j.biopsych.2019.08.010 (PMC7016501; doi:10.1016/j.biopsych.2019.08.010)
Supplement: Supplemental Material [file mmc1.pdf]

Maternal Prenatal Stress Is Associated With Altered Uncinate Fasciculus Microstructure in Premature Neonates

SUPPLEMENTAL INFORMATION

Contents

ADDITIONAL INFORMATION ABOUT THE DATASET 3
Table S1. Focal brain lesions leading to exclusion from subsequent analysis 3
Table S2. Stressful life events scale and equivalent items in the Holmes and Rahe stress scale. 4
Table S3. Diffusion imaging data 6
Table S4. Additional sociodemographic information 7
ADDITIONAL RESULTS 8
Figure S1. 8
Table S5. Relationship between white matter microstructure and predictors in regression models 9
Table S6. Regression analysis for the inferior longitudinal fasciculus 10
Segmentation of uncinate fasciculus in 4 sub-regions 11
Fig S2. 11
Regions of interest for the uncinate fasciculus segmentation 11
Table S8. Relationships between stressful life events and white matter microstructure in ROI1-4. 13
SENSITIVITY ANALYSES 14
a. Imputed data for STAI-TR (n=32) 14
Table S9. Missing answers on the STAI-TR questionnaire according to question number 14
Table S10. Multiple imputations 15
b. Outliers (n=4) 16
Table S11. Stressful life events – Distribution of scores (10 groups) 16
c. Control for postnatal age instead of postmenstrual age 17
d. Controlling for ethnicity 17
e. Multiple births 18
f. Days on ventilation 18
g. Pregnancy complications – Emergency C-section 19
h. Pregnancy complications – Intrauterine growth restriction 19
i. Pregnancy complications – Pregnancy induced hypertension 20
j. Larger sample (n=277) 20
k. GA/PMA range 20
ADDITIONAL BACKGROUND INFORMATION 21

Motivation for selection of uncinate fasciculus and inferior longitudinal fasciculus as tracts of interest 21

Table S12. Relationship between maternal stress and other tracts 23

Additional information on anxiety and stressful life events 24

References 26

ADDITIONAL INFORMATION ABOUT THE DATASET

Table S1. Focal brain lesions leading to exclusion from subsequent analysis

Lesions	n
Periventricular leukomalacia	11
Periventricular leukomalacia with cerebellar hemorrhage / atrophy	1
Hemorrhagic parenchymal infarction	15
Hemorrhagic parenchymal infarction with cerebellar hemorrhage / atrophy	1
Multiple cerebellar hemorrhages or atrophy	5
Thalamic / basal ganglia lesions	1
Other cystic lesion	2
Other hemorrhagic lesion	2
Post hemorrhagic ventricular dilatation	2

Table S2. Stressful life events scale and equivalent items in the Holmes and Rahe stress scale.

Stressful life event	N=	Score	Equivalent to Holmes and Rahe (score)
You were separated or divorced from your partner	13	69	Average of "Divorce"(73) and "Marital separation"(65)
You had a serious illness of injury	16	53	"Major personal injury or illness"(53)
You were physically assaulted	2	53	Used score for "Major personal injury or illness"(53)
You had a miscarriage	41	53	Used score for "You had a serious illness or injury"(53)
A family member or close friend died	44	50	Average of "Death of close family member"(63) and "Death of close friend"(37).
You lost your job	8	47	"Being fired from work"(47)
A family member or close friend was seriously ill	36	44	"Major change in health of family member'(44)
You had treatment for infertility	58	44	Used score for "Major change in health of family member"(44)
Your house was burgled	2	40	No equivalent
You had a major financial problem	20	38	"Major change in financial state"(38)
You were in trouble with the law	0	37	Average of "Detention in jail and "Minor violation of law"(11)
Your partner lost his job	17	37	Used score for "You lost your job" (47) minus 10
Arguments with your partner increased	36	35	"Major change in number of arguments with spouse"(35)
You changed jobs	17	33	Average of "Major business readjustment"(47), "Changing to a different line of work" (36), "Change in responsibilities at work" (29) and "Change in working conditions" (20).

Stressful life event	N=	Score	Equivalent to Holmes and Rahe (score)
You had a serious argument with family or friends	15	29	“In law troubles”(29)
Your partner was in trouble with the law	2	27	Used score for “You were in trouble with the law”(37) minus 10
You moved to a new house/new place to live	67	25	“Major change in living conditions (e.g.new home..) (25)
You had a personal problem at work	22	23	“Troubles with the boss”(23)
You took an examination	12	19	Used score for “Change in usual amount of recreation”(19)
Your partner had problems at work	14	13	Used score for “You had problems at work” minus 10

Table S3. Diffusion imaging data

Diffusion imaging data	Mean (SD)
L-UF FA	0.1648 (0.0158)
L-UF MD	1.3044 (0.0618)
L-UF AD	1.5179 (0.0589)
L-UF RD	1.1977 (0.0645)
R-UF FA	0.1689 (0.0166)
R-UF MD	1.3045 (0.0648)
R-UF AD	1.5276 (0.0610)
R-UF RD	1.1921 (0.0681)
L-ILF FA	0.2069 (0.0221)
L-ILF MD	1.3057 (0.0695)
L-ILF AD	1.5993 (0.0638)
L-ILF RD	1.1589 (0.0749)
R-ILF FA	0.1971 (0.0206)
R-ILF MD	1.3580 (0.0802)
R-ILF AD	1.6448 (0.0791)
R-ILF RD	1.2146 (0.0832)

Abbreviations: L=left, R=right, ILF=inferior longitudinal fasciculus, UF=uncinate fasciculus

Table S4. Additional sociodemographic information

Other infant characteristics	n, %
Mode of delivery	
Emergency caesarean (not in labour)	89, 35.5
Emergency caesarean (in labour)	49, 19.5
Elective caesarean (not in labour)	22, 8.8
Elective caesarean (in labour)	2, 0.8
Vaginal (forceps assisted)	4, 1.6
Vaginal (spontaneous)	83, 33.1
Complications	
Multiple pregnancy	52, 20.7
Pregnancy induced hypertension	48, 19.1
Twin-to-twin transfusion	4, 1.6
Antepartum haemorrhage	34, 13.5
Chorioamnionitis	12, 4.8
Prolonged rupture membranes	43, 17.1
UTI	7, 2.8
Gestational diabetes	12, 4.8
IUGR	45, 17.9
IVF	33, 13.3
Oligohydramnios	18, 7.2
Polyhydramnios	1, 0.4

Abbreviations: UTI=urinary tract infection, IUGR=intrauterine growth restriction, IVF=in vitro fertilisation

ADDITIONAL RESULTS

Figure S1.

Partial regression scatterplots showing the relationships between stressful life events and fractional anisotropy in (A) left and right uncinate fasciculus, (B) left and right inferior frontal longitudinal fasciculus while holding the other predictors constant (i.e. GA, PMA, SES, TPN, maternal age, sex). Points on the scatterplot represent residuals and the regression line includes standard error bars. β=standardized beta, p=significance level before correction for multiple comparisons

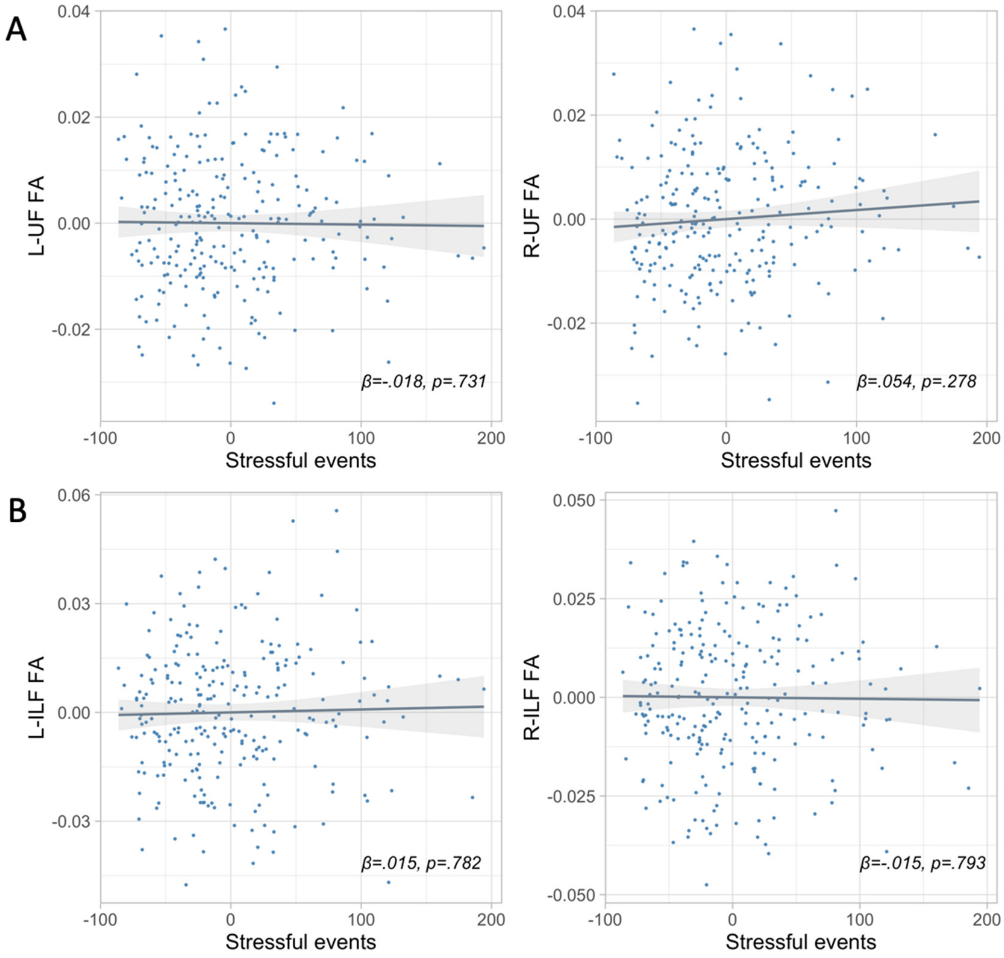

Table S5. Relationship between white matter microstructure and predictors in regression models

		Predictors in multiple regression model							
		Stressful events	STAI-TR	GA	PMA	SES	Maternal age	Days TPN	Sex
L-UF FA	β	-.018	.110	.058	.611	.040	.064	-.070	-.049
	q	.820	.145	.514	<.001*	.545	.452	.467	.500
L-UF MD	β	.149	-.064	.112	-.618	-.054	.017	.071	-.051
	q	.012*	.419	.232	<.001*	.467	.820	.457	.472
L-UF AD	β	.178	-.040	.153	-.533	-.049	.036	-.073	.060
	q	.007*	.545	.098	<.001*	.500	.573	.513	.384
L-UF RD	β	.133	-.073	.091	-.644	-.055	.099	.074	-.040
	q	.026*	.350	.352	<.001*	.457	.915	.452	.525
R-UF FA	β	.054	.045	.012	.650	.087	.035	-.069	-.079
	q	.467	.514	.915	<.001*	.256	.573	.467	.337
R-UF MD	β	.093	-.098	.084	-.642	-.057	-.001	-.306	-.015
	q	.199	.180	.388	<.001*	.457	.981	.500	.838
R-UF AD	β	.142	-.112	.109	-.566	-.033	.003	.054	-.042
	q	.026*	.137	.277	<.001*	.602	.975	.525	.531
R-UF RD	β	.069	-.089	.070	-.663	-.066	-.003	.060	-.002
	q	.366	.220	.457	<.001*	.384	.975	.497	.975

Standardized beta coefficients and significance levels (adjusted for multiple comparisons)

Table S6. Regression analysis for the inferior longitudinal fasciculus

Regression	R^2	Adjusted R^2	F	Stressful events					STAI-TR				
				B	β	T	p	95% CI	B	β	T	p	95% CI
L-ILF FA	.314	.291	13.85	0.000006	.015	.277	.782	-0.000037- 0.000049	0.00014	.068	1.251	.212	-0.00008- 0.00038
R-ILF FA	.262	.238	10.76	-0.000006	-.015	-.262	.793	-0.000047- 0.000036	0.00013	.068	1.205	.229	-0.000087- 0.00036
L-ILF MD	.331	.309	14.98	0.000029	.023	.427	.670	-0.000104- 0.00016	-0.00025	-.038	-703	.483	-0.00098- 0.00046
R-ILF MD	.211	.185	8.08	0.000040	.028	.477	.634	-0.000126- 0.000207	0.000016	-.002	-.028	.978	-0.00091- 0.00089
L-ILF AD	.228	.202	8.919	0.000040	.035	.608	.544	-0.000091- 0.000172	-0.00012	-.020	-.342	.732	-0.00083- 0.00058
R-ILF AD	.141	.113	4.97	0.000039	.027	.452	.651	-0.00013- 0.00021	0.00017	.023	.378	.706	-0.00075- 0.0011
L-ILF RD	.361	.340	17.08	0.000023	.017	.322	.748	-0.00011- 0.00016	-0.00032	-.044	-.840	.402	-0.0010- 0.00043
R-ILF RD	.241	.216	9.58	0.000041	.027	.473	.636	-0.00012- 0.00021	-0.000109	-.013	-.231	.817	-0.0010- 0.00081

Results from multiple regression analyses showing the model fit, as well as associations between stress/anxiety and inferior longitudinal fasciculus microstructural properties. The other covariates in the regression model are gestational age at birth, postmenstrual age at scan, sex, maternal age, socioeconomic status, and days on parenteral nutrition.

Abbreviations: L=left, R=right, ILF=inferior longitudinal fasciculus, ADC=apparent diffusion coefficient, AD=axial diffusivity, RD=radial diffusivity adjusted for GA at birth, PMA at scan, maternal age, SES, number of days on TPN. STAI-TR=State Trait Anxiety Inventory – Trait. B=unstandardized beta, β=standardized beta.

*significant results at p<.05

Segmentation of uncinate fasciculus in 4 sub-regions

The uncinate fasciculus (UF) tract skeleton was manually segmented into 4 sub-regions and vertices on the skeleton surface were labelled as anterior frontal region, insula region 1, insula region II, posterior temporal region (Fig S2).

Fig S2.
Regions of interest for the uncinate fasciculus segmentation viewed as follows (A) left uncinate fasciculus, (B) right uncinate fasciculus. The regions depicted are ROI1 (light blue) anterior frontal region, ROI2 (green) insula region 1, ROI3 (purple) insula region II, ROI4 (dark blue) posterior temporal region.

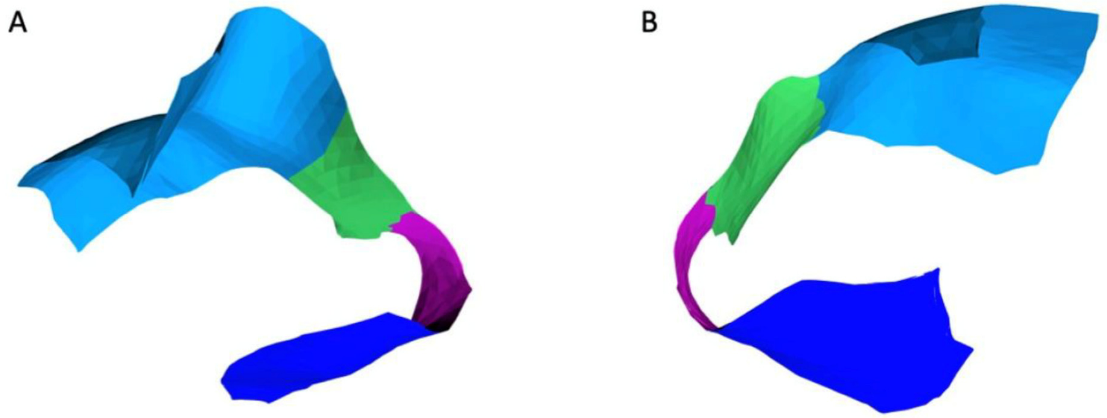

For each subject, AD, RD, MD and FA values were averaged across all vertices within each sub-region (Table S7).

Table S7. Uncinate fasciculus segmentation –Means and standard deviations for individual regions

Side of tract	Characteristic	Region of interest	Mean (SD)
Left	FA	L-UF ROI1	0.1550 (0.0180)
		L-UF ROI2	0.1742 (0.0190)
		L-UF ROI3	0.2254 (0.0215)
		L-UF ROI4	0.1590 (0.1680)
	MD	L-UF ROI1	1.3263 (0.0720)
		L-UF ROI2	1.2282 (0.0579)
		L-UF ROI3	1.1533 (0.0486)
		L-UF ROI4	1.3686 (0.0486)
	AD	L-UF ROI1	1.5272 (0.0675)
		L-UF ROI2	1.4432 (0.0587)
		L-UF ROI3	1.4291 (0.0466)
		L-UF ROI4	1.5884 (0.0953)
	RD	L-UF ROI1	1.2258 (0.0758)
		L-UF ROI2	1.1207 (0.0601)
		L-UF ROI3	1.0154 (0.0529)
		L-UF ROI4	1.2587 (0.0892)
Right	FA	R-UF ROI1	0.1401 (.0206)
		R-UF ROI2	0.1822 (.0201)
		R-UF ROI3	0.2479 (.0223)
		R-UF ROI4	0.1717 (.0182)
	MD	R-UF ROI1	1.3682 (.0805)
		R-UF ROI2	1.3179 (.0588)
		R-UF ROI3	1.0953 (.0453)
		R-UF ROI4	1.2753 (.0919)
	AD	R-UF ROI1	1.5626 (0.0717)
		R-UF ROI2	1.5592 (0.0569)
		R-UF ROI3	1.3957 (0.0478)
		R-UF ROI4	1.4990 (0.0954)
	RD	R-UF ROI1	1.2710 (0.0864)
		R-UF ROI2	1.1973 (0.0628)
		R-UF ROI3	0.0945 (0.0492)
		R-UF ROI4	1.1635 (0.0917)

Abbreviations: L=left, R=right, UF=uncinate fasciculus, FA=fractional anisotropy, MD=mean diffusivity, AD=axial diffusivity, RD=radial diffusivity, ROI1=anterior frontal region, ROI2=insula region 1, ROI3=insula region II, ROI4=posterior temporal region, SD=standard deviation

Exploratory multiple regression analyses were conducted to determine whether scores on stressful life events were associated with white matter microstructure in any particular sub-regions of the UF (RO1 anterior frontal section, ROI2 insula region I, ROI3 insula region II, ROI4 posterior temporal section), controlling for GA, PMA, SES, TPN, sex and maternal age. We focused our analyses on those measures that were significantly associated with maternal stressful life events when assessing the whole tract (axial, radial and mean diffusivity of left UF and axial diffusivity of the right UF).

There were significant relationships between UF microstructure and stressful life events for regions 1-3, although only values for left ROI1, anterior frontal section, retained significance after correction for multiple comparisons (left AD: β=.182, p=.001, q=.007; left MD: β=.150, p=.004, q=.027) (Table S8).

Of note, previous studies have reported white matter abnormality in the anterior section of the UF in generalized anxiety disorder (Phan et al., 2009) and sensitivity to early-life stress (Ho et al., 2017).

Table S8. Relationships between stressful life events and white matter microstructure in ROI1-4.

		Model R^2	Stress		
			β	p**	q
ROI1	L-UF AD	.272	.182	.001*	.007
	L-UF RD	.361	.132	.011	.058
	L-UF MD	.339	.150	.004*	.027
	R-UF AD	.305	.125	.020	.092
ROI2	L-UF AD	.320	.122	.023	.092
	L-UF RD	.411	.111	.026	.092
	L-UF MD	.401	.118	.019	.091
	R-UF AD	-	-	-	-
ROI3	L-UF AD	.276	.110	.045	.146
	L-UF RD	-	-	-	-
	L-UF MD	.379	.105	.040	.097
	R-UF AD	.260	.128	.022	.092
ROI4	L-UF AD	-	-	-	-
	L-UF RD	-	-	-	-
	L-UF MD	-	-	-	-
	R-UF AD	-	-	-	-

*values that survived correction for multiple comparisons

** for clarity, only those values that were significant at p<.05 before correction for multiple comparisons are presented.

SENSITIVITY ANALYSES

Please note that throughout this paper, R^2 refers to the whole model.

a. Imputed data for STAI-TR (n=32)

Table S9. Missing answers on the STAI-TR questionnaire according to question number.

Question number	Number missing / 251
**15**	**8**
**1**	**6**
**16**	**4**
**12**	**3**
**14**	**2**
**8**	**2**
**19**	**2**
**6**	**2**
**4**	**1**
**7**	**1**
**2**	**1**
**13**	**1**
**20**	**1**
**6**	**1**
**10**	**1**
**18**	**1**

We conducted independent samples t-tests to investigate whether there are any differences in demographics between cases with and without missing values on the STAI-TR. There was no significant difference in stressful life events scores, GA, PMA, birth weight, maternal age or days on parenteral nutrition. There was a significant difference in socioeconomic status t(249)=-2.227, p=.030, with higher scores on the index of multiple deprivation for women with missing values (mean(SD)=23.83(11.93)) than without missing values (mean(SD)=18.75(12.05)). There was no significant difference in uncinate fasciculus microstructure between the 2 groups.

We then repeated our main analysis on n=219, removing the n=32 cases where missing values were imputed for STAI-TR. The pattern of results remained the same. More specifically, after correcting for multiple comparisons, stressful life events was associated with L UF MD (R^2=.45, β=.165, q=.006) , L UF AD (R^2=.39, β=.194, q=.002)., L UF RD (R^2=.47, β=.149, q=.019) and R-UF AD (R^2=.41, β=.156,

q=.019). There were no significant relationships between uncinate fasciculus microstructure and trait anxiety.

Further, we repeated our analysis using multiple imputation with 5 imputations and compared this with our results obtained from imputation using the mean. For all cases, the median was 36.00 and the range was 20.00-68.00.

Table S10. Multiple imputations

	**Mean imp**	**1st imp**	**2nd imp**	**3rd imp**	**4th imp**	**5th imp**
Mean<br>(S.D)	38.02<br>(10.15)	38.14<br>(10.08)	38.09<br>(10.09)	38.09<br>(10.08)	38.11<br>(10.07)	38.10<br>(10.08)
L-UF FA<br>Stress<br>STAI	R^2=.37<br>-<br>p=.034	R^2=.37<br>-<br>p=.026	R^2=.37<br>-<br>p=.027	R^2=.37<br>-<br>p=.026	R^2=.37<br>-<br>p=.030	R^2=.37<br>-<br>p=.026
L-UF MD<br>Stress<br>STAI	R^2=.44<br>p=.002*<br>-	R^2=.44<br>p=.002*<br>-	R^2=.44<br>p=.002*<br>-	R^2=.44<br>p=.002*<br>-	R^2=.44<br>p=.002*<br>-	R^2=.44<br>p=.002*<br>-
L-UF AD<br>Stress<br>STAI	R^2=.37<br>p=.001*<br>-	R^2=.37<br>p=.001*<br>-	R^2=.37<br>p=.001*<br>-	R^2=.37<br>p=.001*<br>-	R^2=.37<br>p=.001*<br>-	R^2=.37<br>p=.001*<br>-
L-UF RD<br>Stress<br>STAI	R^2=.46<br>p=.005*<br>-	R^2=.46<br>p=.005*<br>-	R^2=.46<br>p=.005*<br>-	R^2=.46<br>p=.005*<br>-	R^2=.46<br>p=.005*<br>-	R^2=.46<br>p=.005*<br>-
R-UF FA<br>Stress<br>STAI	R^2=.41<br>-<br>-	R^2=.41<br>-<br>-	R^2=.41<br>-<br>-	R^2=.41<br>-<br>-	R^2=.41<br>-<br>-	R^2=.41<br>-<br>-
R-UF MD<br>Stress<br>STAI	R^2=.45<br>-<br>p=.045	R^2=.45<br>-<br>p=.044	R^2=.45<br>-<br>p=.044	R^2=.45<br>-<br>p=.042	R^2=.45<br>-<br>p=.049	R^2=.45<br>-<br>p=.042

	**Mean imp**	**1st imp**	**2nd imp**	**3rd imp**	**4th imp**	**5th imp**
R-UF AD<br>Stress<br>STAI	R^2=.39<br>p=.005*<br>p=.030	R^2=.39<br>p=.005*<br>p=.032	R^2=.39<br>p=.005*<br>p=.032	R^2=.39<br>p=.005*<br>p=.031	R^2=.39<br>p=.005*<br>p=.034	R^2=.39<br>p=.005*<br>p=.030
R-UF RD<br>Stress<br>STAI	R^2=.47<br>-<br>-	R^2=.47<br>-<br>-	R^2=.47<br>-<br>-	R^2=.47<br>-<br>-	R^2=.47<br>-<br>-	R^2=.47<br>-<br>-

For simplicity, only values with p<.05 are presented. *=survived correction for multiple comparisons using FDR correction.

b. Outliers (n=4)

Table S11. Stressful life events – Distribution of scores (10 groups)

**Score**	**n**
0	36
1-30	37
31-60	65
61-90	32
91-120	42
121-150	16
151-180	12
181-210	7
211-240	1
241-270	3

By examining scatterplots, we identified 4 outliers (cases 74, 83, 218 and 220), with high scores on the measure of stressful life events (scores 264, 270, 255 and 236). These cases were kept in the main analysis as they represent real data rather than a measurement error. However, we repeated our analyses

on n=247, removing these 4 outliers. The pattern of results remained largely the same. More specifically, after correcting for multiple comparisons, stressful life events was associated with L-UF MD (R^2=.43, β=.152, q=.032), L-UF AD (R^2=.37, β=.185, q=.006), R-UF AD (R^2=.40, β=.173, q=.011). The relationship between stressful life events and L-UF RD did not survive correction for multiple comparisons (R^2=.45, β=.134, q=.082). There were no significant relationships between uncinate fasciculus microstructure and trait anxiety.

c. Control for postnatal age instead of postmenstrual age

Gestational age and postnatal age correlated very highly (r=-.85, p<.001) and introducing them in the same model would cause issues with multicollinearity. Meanwhile, the correlation between GA and PMA was only r=-.24, p<.001 and thus PMA was included in the main analysis.

However, we have repeated our analyses controlling for postnatal age instead of postmenstrual age. The pattern of results remained largely the same. More specifically, after correcting for multiple comparisons, stressful life events was associated with L-UF MD (R^2=.44, β=.153, q=.007), L-UF AD (R^2=.36, β=.181, q=.003) L-UF RD (R2=.46, β=.137, q=.016). The relationship between stressful life events and R-UF AD did not survive correction for multiple comparisons (R^2=.37, β=.146, q=.054). There were no significant relationships between uncinate fasciculus microstructure and trait anxiety.

d. Controlling for ethnicity

We repeated our analyses introducing maternal ethnicity in the model to see whether this would influence the results. The pattern of results remained the same. More specifically, after correcting for multiple comparisons, stressful life events was associated with L UF MD (R^2=.44, β=.153, q=.014), L UF AD (R^2=.37, β=.179, q=.008), L UF RD (R^2=.47, β=.149, q=.026) and R-UF AD (R^2=.39, β=.141, q=.042). There were no significant relationships between uncinate fasciculus microstructure and trait anxiety.

e. Multiple births

We repeated our analysis with the other twin (or one of the siblings in case of triplets) included in the sample. The pattern of results remained the same. After correcting for multiple comparisons, stressful life events was associated with L UF MD (R^2=.42, β=.146, q=.019), L UF AD (R^2=.35, β=.177, q=.007), L UF RD (R^2=.44, β=.129, q=.048), while R-UF AD did not survive correction for multiple comparisons (R^2=.34, β=.133, q=.054, p value before correction=.012). There were no significant relationships between uncinate fasciculus microstructure and trait anxiety.

We repeated our analysis introducing multiples (no=singleton birth, yes=multiple birth) as a predictor in the model. The pattern of results remained the same. More specifically, after correcting for multiple comparisons, stressful life events was associated with L UF MD (R^2=.44, β=.148, q=.021), L UF AD (R^2=.37, β=.175, q=.008), L UF RD (R^2=.46, β=.133, q=.036) and R-UF AD (R^2=.39, β=.147, q=.032). There were no significant relationships between uncinate fasciculus microstructure and trait anxiety.

Then, we excluded all the multiple births (n=52) and repeated the main analysis on singleton cases only (n=199). After correcting for multiple comparisons, stressful life events was associated with L UF AD (R^2=.35, β=.169, q=.035) and R-UF AD (R^2=.36, β=.159, q=.044). As expected given the smaller sample size, the relationship between stressful life events and L-UF MD (R^2=.42, β=.138, q=.142) and L-UF RD (R^2=.44, β=.121, q=.169) did not survive correction for multiple comparisons. There were no significant relationships between uncinate fasciculus microstructure and trait anxiety.

f. Days on ventilation

"Days on total parenteral nutrition" was included in the model over "days on ventilation" based on the distribution of scores (Days TPN: median=6, range 0-59; Days Ventilation: median=0, range 0-33).

However, we repeated our analysis including days on ventilation in the model, instead of days on TPN, to see whether this change would affect the results. The pattern of results remained the same. More specifically, after correcting for multiple comparisons, stressful life events was associated with L UF

MD (R^2=.44, β=.151, q=.012), L UF AD (R^2=.37, β=.181, q=.007), L UF RD (R^2=.46, β=.133, q=.034) and R-UF AD (R^2=.39, β=.140, q=.037). There were no significant relationships between uncinate fasciculus microstructure and trait anxiety.

g. Pregnancy complications – Emergency C-section

We repeated our analysis introducing emergency C-section (yes/no) as a predictor in the model. The pattern of results remained the same. More specifically, after correcting for multiple comparisons, stressful life events was associated with L UF MD (R^2=.44, β=.148, q=.021), L UF AD (R^2=.37, β=.175, q=.008), L UF RD (R^2=.46, β=.133, q=.026) and R-UF AD (R^2=.39, β=.147, q=.026). There were no significant relationships between uncinate fasciculus microstructure and trait anxiety.

Then, we excluded all cases with emergency c-section (n=138). Stressful life events was associated with L UF MD (R^2=.44, β=.148, q=.093), L UF AD (R^2=.37, β=.178, q=.069), L UF RD (R^2=.46, β=.132, q=.124) and R-UF AD (R^2=.39, β=.147, q=.069). The p values before correction for multiple comparisons were p=.013, p=.012, p=.022 and p=.012 respectively, but, as expected given the small sample size, none of these values survived correction for multiple comparisons.

h. Pregnancy complications – Intrauterine growth restriction

We repeated our analysis excluding cases with intrauterine growth restriction (n=45). After correcting for multiple comparisons, stressful life events was associated with L UF AD (R^2=.37, β=.194, q=.007), L-UF MD (R^2=.44, β=.171, q=.012) and L-UF RD (R^2=.46, β=.158, q=.017). The relationship between stressful life events and R-UF AD (R^2=.38, β=.135, q=.044) did not survive correction for multiple comparisons. There were no significant relationships between uncinate fasciculus microstructure and trait anxiety.

i. Pregnancy complications – Pregnancy induced hypertension

We repeated our analysis introducing pregnancy induced hypertension (yes/no) as a predictor in the model. The pattern of results remained the same. More specifically, after correcting for multiple comparisons, stressful life events was associated with L UF MD (R^2=.44, β=.154, q=.014), L UF AD (R^2=.38, β=.186, q=.002), L UF RD (R^2=.46, β=.136, q=.03) and R-UF AD (R^2=.40, β=.153, q=.019). There were no significant relationships between uncinate fasciculus microstructure and trait anxiety.

Then, we excluded all cases with pregnancy induced hypertension (n=48). Stressful life events was associated with L UF MD (R^2=.48, β=.127, q=.093), L UF AD (R^2=.39, β=.145, q=.076), L UF RD (R^2=.51, β=.116, q=.114) and R-UF AD (R^2=.44, β=.128, q=.10). The p values before correction for multiple comparisons were p=.016, p=.012, p=.024, p=.020 respectively, but none of these values survived correction for multiple comparisons.

j. Larger sample (n=277)

We repeated our analysis with a larger sample, which included all participants who had completed the stressful life events questionnaire, regardless of the data availability for the STAI-TR questionnaire. STAI-TR was not included in the model. The pattern of results remained the same. More specifically, after correcting for multiple comparisons, stressful life events was associated with L UF MD (R^2=.43, β=.134, q=.022), L UF AD (R^2=.37, β=.160, q=.006), L UF RD (R^2=.45, β=.120, q=.042) and R-UF AD (R^2=.38, β=.135, q=.030). There were no significant relationships between uncinate fasciculus microstructure and trait anxiety.

k. GA/PMA range

As our sample includes both a large range for gestational age at birth (23.57-32.86) as well as a large range for postmenstrual age at scan (37.86-45.71), we accounted for this in additional analyses.

First, we repeated our analysis including all gestational ages, but restricting the analysis to those scanned at PMA 38.00-43.00. The remaining sample was n=160 and the pattern of results remained largely the same. More specifically, after correcting for multiple comparisons, stressful life events was associated with L UF AD (R^2=.31, β=.199, q=.025) and R-UF AD (R^2=.31, β=.210, q=.014). The relationship between stressful life events and L UF MD approached significance (R^2=.36, β=.172, q=.052, p value before correction=.009). The relationship between stressful life events and L UF RD did not survive correction for multiple comparisons (R^2=.38, β=.157, q=.085, p value before correction=.016). There were no significant relationships between uncinate fasciculus microstructure and trait anxiety.

Secondly, we repeated our analysis including all postmenstrual ages but restricting our analysis to those born very preterm (GA 28+). We excluded n=56 infants born extremely preterm. The remaining sample was n=195. The results did not survive correction for multiple comparisons. More specifically, stressful life events was associated with L UF MD (R^2=.46, β=.120, q=.12, p=.029) , L UF AD (R^2=.36, β=.140, q=.10, p=.019), L UF RD (R^2=.49, β=.108, q=.14, p=.042) and R-UF AD (R^2=.42, β=.126, q=.11, p=.026).

ADDITIONAL BACKGROUND INFORMATION

Motivation for selection of uncinate fasciculus and inferior longitudinal fasciculus as tracts of interest

The uncinate fasciculus (UF) was chosen based on previous literature relating abnormalities in its microstructure to a wide range of neurodevelopmental and psychiatric disorders, as well as to exposure to stressful events, as detailed below:

Prenatal stress exposure has been linked to abnormal neurodevelopment of a number of brain regions including the limbic system and prefrontal cortex, in both animal (Uno et al. 1994; Salm et al. 2004; Kraszpulski et al. 2006; Tamura et al. 2011) and human studies (e.g. Qiu et al., 2013, Buss et al., 2010). The UF connects areas of the limbic system with the frontal lobes, and it has been suggested that limbic regions are particularly vulnerable to prolonged stress during early development (Olson et al., 2015),

possibly due to excitotoxicity from glucocorticoids (Conrad et al., 2008). Abnormalities in functional connectivity between limbic and frontal areas have also been widely reported in anxiety disorders (Etkin et al., 2009, Etkin et al., 2010). Thus, in order to investigate whether maternal prenatal stress is associated with altered structural connectivity between the limbic system and frontal lobes, we focused specifically on the microstructural properties of the uncinate fasciculus. Abnormalities in UF microstructure are commonly reported in anxiety disorders and early-life stress (Phan et al., 2009, Hettema et al., 2012, Tromp et al., 2012, Costanzo et al., 2016, Koch et al., 2017). In a study of young adults, Hanson et al. (2015) reported reduced structural integrity of the UF in those who experienced childhood maltreatment, while in a study of early adolescence, higher sensitivity to early life stress was associated with altered microstructure of the uncinate fasciculus, which predicted higher levels of anxiety symptomatology (Ho et al., 2017).

The inferior longitudinal fasciculus (ILF) connects the occipital cortex to the temporal lobe (Ashtari et al., 2011) and alterations in ILF integrity have mostly been reported in relation to impaired visual processing and object naming (Ortibus et al., 2012, Shinouara et al., 2010, Shinouara et al., 2007), but also schizophrenia (Ashtari et al., 2007) and autism spectrum conditions (Koldewyn et al., 2014). To our knowledge, no research studies have implicated the ILF in the development of anxiety disorders in children. Our decision to include the ILF as a control tract was based on previous literature which included the ILF as a control tract in studies focusing on the UF (Sarkar et al., 2014). We chose to focus on 2 tracts to avoid multiple comparisons issues. A control tract was used to ensure that the findings did not stem from an overall global white matter impairment, but were specific to the uncinate fasciculus.

We repeated our analyses with other control tracts: corpus callosum, left and right superior longitudinal fasciculus, and left and right inferior fronto-occipital fasciculus. There was no significant relationship between FA, MD, RD and AD in these tracts and either stressful life events or maternal trait anxiety (no p values $<.05$ even before correction for multiple comparisons), with the exception of STAI-TR and L-IFO (Beta=.106, p=.024). However, this did not survive correction for multiple comparisons (Table S12).

The UF and the ILF are late-developing association fibres (Lebel et al., 2012). The UF and ILF have similar developmental trajectories in the fetal and early postnatal period, with myelination commencing in the 3rd postnatal month (Yakolev & Lecours, 1967; Hasegawa, 1992; Gilles 1983, Dubois et al., 2014). Thus, the differences observed in this study are unlikely to occur as a result of differences in myelination. The neural mechanisms underlying the association between PNSE to UF development are unclear and we are not able to assess mechanisms in this observational study.

The neural mechanisms underlying the association between PNSE to UF development are unclear. Maternal anxiety is associated with physiological changes including alterations in uterine blood flow (Fisk & Glover, 1999), cortisol production (Risbrough & Stein, 2006, Staufenbiel et al., 2013, Abelson et al., 2007) and fetal heart rate (Monk et al., 2000). Indeed, a recent study assessing structural and functional connectivity in infants exposed to maternal depression suggested that alterations in fetal heart rate may influence the development of the amygdala-prefrontal circuit (Posner et al, 2016). However, we do not have fetal physiological data in these infants or data regarding maternal cortisol levels, and we are not able to disentangle genetic versus environmental influences of uncinate fasciculus development.

Table S12. Relationship between maternal stress and other tracts

**Tract**		**Stressful life events (p value)**	**STAI-TR (p value)**
Corpus callosum	FA	.811	.397
	MD	.439	.933
	AD	.352	.608
	RD	.489	.922
L-CST	FA	.891	.069
	MD	.685	.616
	AD	.638	.812
	RD	.716	.421
L-IFO	FA	.406	.024
	MD	.308	.496

Tract		Stressful life events (p value)	STAI-TR (p value)
	AD	.338	.999
	RD	.313	.336
L-SLF	FA	.485	.071
	MD	.379	.396
	AD	.374	.752
	RD	.393	.283
R-CST	FA	.898	.171
	MD	.220	.978
	AD	.099	.640
	RD	.314	.800
R-IFO	FA	.951	.051
	MD	.229	.320
	AD	.147	.602
	RD	.298	.240
R-SLF	FA	.626	.103
	MD	.456	.904
	AD	.470	.699
	RD	.465	.723

L=left, R=right, CST=corticospinal tract, IFO=inferior fronto-occipital fasciculus, SLF=superior longitudinal fasciculus

Additional information on anxiety and stressful life events

On the STAI-TR a cut-off of 40 provides a high degree of accuracy in identifying women with a diagnosed anxiety disorder (Sinesi et al., 2019). In our sample, on the STAI-TR, n=97 women scored 40 or over (n=64 scored 40-49, n=24 scored 50-59, n=9 scored 60-69) and n=154 scored under 40 (n=56 scored 20-29, n=98 sored 30-39). In our sample, maternal trait anxiety scores are low (median 36) and well below clinical cut-offs (Dennis et al., 2013, Grant et al., 2008). Previous studies reporting associations between maternal antenatal anxiety and infant brain development have focused on state, rather than trait anxiety (Dean et al., 2018) or a combined score of state and trait (Rifkin-Graboi et al.,

2015) and included participants with higher levels of anxiety (e.g. threshold ~43, Rifkin-Graboi et al., 2015). To our knowledge, only one study (Qiu et al., 2013) examined the relationship between maternal trait anxiety and infant brain development, and reported no significant association between trait anxiety and hippocampal volume at birth.

Trait anxiety, as measured using the STAI-TR, is seen as a relatively stable personality trait (Spielberger, 1972), which implies a generalized and enduring predisposition to respond to situations in an anxious manner. Dennis et al (2013) examined the stability of STAI scores in the perinatal period and reported that overall STAI scores at 1 week postpartum were significantly correlated to 4-week and 8-week scores. Further, high scores on STAI-ST have been reported to occur at similar rates between the third trimester (18.2%) and childbirth (18.6%); scores for STAI-TR were not reported in this study (Figuereido et al., 2011). Lastly, Grant et al (2008) reported considerable stability in self-reported anxiety between pregnancy and 7 months postpartum, arguing that trait anxiety persists from pregnancy into the postnatal period, rather than representing a transient state.

Several studies have investigated the reliability of maternal recall for pregnancy and birth related events (Sou et al., 2006, Tomeo et al., 1999, Quigley et al. 2007). For example, in the study by Tomeo et al. (1999), recall of pregnancy-related events was highly accurate when comparing self-report measures collected approximately 30 years after birth to data collected during pregnancy. Further, the agreement between hospital records and maternal reporting of mode of delivery retrospectively assessed at 9 months postpartum was 94% (Quigley et al. 2007). To our knowledge, no studies have examined the reliability of maternal recall specifically for adverse life events. However, studies reporting on the validity of retrospective reports of adverse events have suggested that false positive reports are rare (Hardt et al., 2004).

References

Abelson JL, Khan S, Liberzon I & Young EA. (2007): HPA axis activity in patients with panic disorder: review and synthesis of four studies. *Depression and anxiety, 24*(1), 66-76.

Ashtari M, Cottone J, Ardekani BA, Cervellione K, Szeszko PR, Wu J et al., (2007). Disruption of white matter integrity in the inferior longitudinal fasciculus in adolescents with schizophrenia as revealed by fiber tractography*. Archives of general psychiatry, 64*(11), pp.1270-1280.

Ashtari M. (2011): Anatomy and functional role of the inferior longitudinal fasciculus: a search that has just begun. *Dev Med Child Neurol 54*, 6–7.

Buss C, Davis EP, Muftuler LT, Head K, Sandman, CA, & Sandman CA (2010): High pregnancy anxiety during mid-gestation is associated with decreased gray matter density in 6—9-year-old children. *Psychoneuroendocrinology, 35*(1), 141–153.

Conrad CD. (2008): Chronic stress-induced hippocampal vulnerability: the glucocorticoid vulnerability hypothesis. *Reviews in the Neurosciences, 19*(6), 395-412.

Costanzo ME, Jovanovic T, Pham D, Leaman S, Highland KB, Norrholm SD et al. (2016): White matter microstructure of the uncinate fasciculus is associated with subthreshold posttraumatic stress disorder symptoms and fear potentiated startle during early extinction in recently deployed service members. *Neuroscience letters, 618*, 66-71.

Dean DC, Planalp EM, Wooten W, Kecskemeti SR, Adluru N, Schmidt CK et al. (2018): Association of prenatal maternal depression and anxiety symptoms with infant white matter microstructure. *JAMA pediatrics, 172*(10), 973-981.

Dennis CL, Coghlan M. and Vigod S (2013): Can we identify mothers at-risk for postpartum anxiety in the immediate postpartum period using the State-Trait Anxiety Inventory? *Journal of Affective Disorders, 150*(3), pp.1217-1220.

Dubois J, Dehaene-Lambertz G, Kulikova S, Poupon C, Hüppi PS, and Hertz-Pannier L (2014): The early development of brain white matter: a review of imaging studies in fetuses, newborns and infants. *Neuroscience, 276*, pp.48-71.

Etkin A, Prater KE, Schatzberg AF, et al. (2009): Disrupted amygdalar subregion functional connectivity and evidence of a compensatory network in generalized anxiety disorder. *Arch Gen Psychiatry;*66:1361–72.

Etkin A, Prater KE, Hoeft F, Menon V, & Schatzberg AF. (2010): Failure of anterior cingulate activation and connectivity with the amygdala during implicit regulation of emotional processing in generalized anxiety disorder. *American Journal of Psychiatry, 167*(5), 545-554.

Figueiredo B. and Conde A. (2011): Anxiety and depression in women and men from early pregnancy to 3-months postpartum*. Archives of women's mental health, 14*(3), pp.247-255.

Fisk NM, & Glover V. (1999): Association between maternal anxiety in pregnancy and increased uterine artery resistance index: cohort based study. *Bmj, 318*(7177), 153-157.

Gilles FH, Shankle W, & Dooling EC. (1983): Myelinated tracts: growth patterns. *In The developing human brain* (pp. 117-183). Butterworth-Heinemann.

Grant KA, McMahon C. and Austin MP (2008): Maternal anxiety during the transition to parenthood: a prospective study*. Journal of affective disorders, 108(*1-2), pp.101-111.

Hanson JL, Knodt AR, Brigidi BD, & Hariri AR. (2015): Lower structural integrity of the uncinate fasciculus is associated with a history of child maltreatment and future psychological vulnerability to stress*. Development and psychopathology, 27*(4pt2), 1611-1619.

Hardt J. and Rutter M. (2004): Validity of adult retrospective reports of adverse childhood experiences: review of the evidence. *Journal of child psychology and psychiatry, 45*(2), pp.260-273.

Hasegawa M, Houdou S, Mito T, Takashima S, Asanuma K, & Ohno T. (1992): Development of myelination in the human fetal and infant cerebrum: a myelin basic protein immunohistochemical study. *Brain and Development, 14*(1), 1-6.

Hettema JM, Kettenmann B, Ahluwalia V, McCarthy C, Kates WR, Schmitt JE. Et al. (2012). Pilot multimodal twin imaging study of generalized anxiety disorder. *Depression and anxiety, 29*(3), 202-209.

Ho TC, King LS, Leong JK, Colich NL, Humphreys KL, Ordaz SJ et al. (2017): Effects of sensitivity to life stress on uncinate fasciculus segments in early adolescence. *Social cognitive and affective neuroscience, 12*(9), 1460-1469.

Koch SB, Van Zuiden M, Nawijn L, Frijling JL, Veltman DJ, & Olff M. (2017): Decreased uncinate fasciculus tract integrity in male and female patients with PTSD: a diffusion tensor imaging study. *Journal of psychiatry & neuroscience: JPN,* 42(5), 331.

Koldewyn K, Yendiki A, Weigelt S, Gweon H, Julian J, Richardson H. et al. (2014): Differences in the right inferior longitudinal fasciculus but no general disruption of white matter tracts in children with autism spectrum disorder. *Proceedings of the National Academy of Sciences, 111*(5), pp.1981-1986.

Kraszpulski M, Dickerson PA, & Salm AK. (2006): Prenatal stress affects the developmental trajectory of the rat amygdala. *Stress, 9*(2), 85-95.

Lebel C, Gee M, Camicioli R, Wieler M, Martin W. and Beaulieu C. (2012): Diffusion tensor imaging of white matter tract evolution over the lifespan. *Neuroimage, 60*(1), pp.340-352.

Monk C, Fifer WP, Myers MM, Sloan RP, Trien L, & Hurtado A. (2000): Maternal stress responses and anxiety during pregnancy: effects on fetal heart rate. *Developmental Psychobiology: The Journal of the International Society for Developmental Psychobiology, 36*(1), 67-77.

Olson IR, Von Der Heide RJ, Alm KH, & Vyas G. (2015): Development of the uncinate fasciculus: implications for theory and developmental disorders. *Developmental cognitive neuroscience, 14*, 50-61.

Ortibus ELS, Verhoeven J, Sunaert S, Casteels I, De Cock P. and Lagae L. (2012): Integrity of the inferior longitudinal fasciculus and impaired object recognition in children: a diffusion tensor imaging study. *Developmental Medicine & Child Neurology, 54*(1), pp.38-43.

Phan KL, Orlichenko A, Boyd E, Angstadt M, Coccaro EF, Liberzon I et al. (2009): Preliminary evidence of white matter abnormality in the uncinate fasciculus in generalized social anxiety disorder. *Biological psychiatry, 66*(7), 691-694.

Posner J, Cha J, Roy AK, Peterson BS, Bansal R, Gustafsson HC et al. (2016): Alterations in amygdala–prefrontal circuits in infants exposed to prenatal maternal depression. *Translational psychiatry, 6*(11), e935.

Qiu A, Rifkin-Graboi A, Chen H, Chong YS, Kwek K, Gluckman P. et al. (2013): Maternal anxiety and infants' hippocampal development: timing matters. *Translational Psychiatry, 3*

Quigley MA, Hockley C. and Davidson LL. (2007): Agreement between hospital records and maternal recall of mode of delivery: evidence from 12 391 deliveries in the UK Millennium Cohort Study. BJOG: *An International Journal of Obstetrics & Gynaecology, 114*(2), pp.195-200.

Rifkin-Graboi A, Meaney MJ, Chen H, Bai J, Bak W, Thway Tint M et al. (2015): Antenatal Maternal Anxiety Predicts Variations in Neural Structures Implicated in Anxiety Disorders in Newborns. *Journal of the American Academy of Child & Adolescent Psychiatry, 54*(4), 313–321

Risbrough VB, & Stein MB. (2006): Role of corticotropin releasing factor in anxiety disorders: a translational research perspective. *Hormones and behavior, 50*(4), 550-561

Salm AK, Pavelko M, Krouse EM, Webster W, Kraszpulski M, & Birkle DL. (2004): Lateral amygdaloid nucleus expansion in adult rats is associated with exposure to prenatal stress. *Developmental brain research, 148*(2), 159-167.

Sarkar S, Craig MC, Dell'Acqua F, O'Connor TG, Catani M, Deeley Q et al. (2014): Prenatal stress and limbic-prefrontal white matter microstructure in children aged 6–9 years: a preliminary diffusion tensor imaging study. *The World Journal of Biological Psychiatry, 15*(4), 346-352.

Shinoura N, Suzuki Y, Tsukada M, Katsuki S, Yamada R, Tabei Y et al. (2007). Impairment of inferior longitudinal fasciculus plays a role in visual memory disturbance. *Neurocase, 13*(2), pp.127-130.

Shinoura N, Suzuki Y, Tsukada M, Yoshida M, Yamada R, Tabei Y. et al. (2010): Deficits in the left inferior longitudinal fasciculus results in impairments in object naming. *Neurocase, 16*(2), pp.135-139.

Sinesi A, Maxwell M, O'Carroll R. and Cheyne H. (2019): Anxiety scales used in pregnancy: systematic review. *BJPsych open, 5*(1).

Sou SC, Chen WJ, Hsieh WS. and Jeng SF. (2006): Severe obstetric complications and birth characteristics in preterm or term delivery were accurately recalled by mothers. *Journal of clinical epidemiology, 59*(4), pp.429-435.

Spielberger CD (1972): Anxiety as an emotional state. *Anxiety-Current trends and theory,* 3-20.

Staufenbiel SM, Penninx BW, Spijker AT, Elzinga BM, & van Rossum EF. (2013): Hair cortisol, stress exposure, and mental health in humans: a systematic review. *Psychoneuroendocrinology, 38*(8), 1220-1235.

Tamura M, Sajo M, Kakita A, Matsuki N, & Koyama R. (2011): Prenatal stress inhibits neuronal maturation through downregulation of mineralocorticoid receptors. *Journal of Neuroscience, 31*(32), 11505-11514.

Tomeo CA, Rich-Edwards JW, Michels KB, Berkey CS, Hunter DJ, Frazier AL et al. (1999): Reproducibility and validity of maternal recall of pregnancy-related events. *Epidemiology,* pp.774-777.

Tromp DP, Grupe DW, Oathes DJ, McFarlin DR, Hernandez PJ, Kral TR (2012): Reduced structural connectivity of a major frontolimbic pathway in generalized anxiety disorder. *Archives of general psychiatry, 69*(9), 925-934

Uno H, Eisele S, Sakai A, Shelton S, Baker E, DeJesus O. (1994): Neurotoxicity of glucocorticoids in the primate brain. *Hormones and behavior, 28(*4), 336-348.

Yakolev PI. & LeCours AR. (1967): The myelogenetic cycles of regional maturation of the brain. *Regional development of the Brain in early life,* Mikowski.
